# The Effectiveness of Ondansetron and Dexamethasone in Preventing Postoperative Nausea and Vomiting After Laparoscopic Cholecystectomy

**DOI:** 10.7759/cureus.37419

**Published:** 2023-04-11

**Authors:** Farzad Qasemi, Tahmina Aini, Wahida Ali, Wahidullah Dost, Mohammad Qaher Rasully, Maiwand Anwari, Wahida Dost, Rabia Zaheer, Raisa Dost, Abdul Subhan Talpur

**Affiliations:** 1 General Surgery, Dr. Ruth K. M. Pfau, Civil Hospital, Karachi, PAK; 2 General Surgery, Jamhuriat Hospital, Kabul, AFG; 3 General Surgery, Jinnah Postgraduate Medical Center, Karachi, PAK; 4 Internal Medicine, Liaquat University of Medical and Health Sciences, Jamshoro, PAK; 5 Medicine, Liaquat University of Medical and Health Sciences, Jamshoro, PAK

**Keywords:** corticosteroid treatment, laparoscopic cholecystectomy, dexamethasone, ondansetron, postoperative nausea vomiting

## Abstract

Background

The current research compared the effectiveness of dexamethasone with ondansetron in terms of the frequency of postoperative nausea and vomiting in patients undergoing laparoscopic cholecystectomy.

Methodology

A comparative cross-sectional study was conducted in the Department of Surgery, Civil Hospital, Karachi, Pakistan, between June 2021 and March 2022. All patients aged between 18 and 70 years who were scheduled for elective laparoscopic cholecystectomy under general anesthesia were included in the study. All women who were on antiemetics or cortisone before surgery pregnant, and had hepatic, or renal malfunction were excluded. Group A included patients who were administered 8 mg of dexamethasone intravenously, and group B included patients who were prescribed 4 mg of ondansetron intravenously. Observation of patients was done for any symptoms such as vomiting, nausea, or the need for any antiemetic medication after the surgery. The number of episodes of vomiting and nausea was recorded in the proforma along with the duration of stay in the hospital.

Results

A total of 259 patients were examined during the study - 129 (49.8%) in the dexamethasone group (group A) and 130 (50.2%) in the ondansetron group (group B). The mean age of group A was 42.56 ± 11.9 years, with a mean weight of 61.4 ± 8.5 kg. The mean age of group B was 41.19 ± 10.8 years, with a mean weight of 62.56 ± 6.3 kg. Upon assessing the effectiveness of each drug in preventing nausea and vomiting, postoperatively, it was found that both drugs were equally effective in preventing nausea in the majority of the patients (73.85% vs. 65.89%; *P *= 0.162). However, ondansetron was significantly more effective in preventing vomiting in patients than dexamethasone (91.54% vs. 79.07%; *P *= 0.004).

Conclusions

This study concluded that the use of either dexamethasone or ondansetron effectively reduces the incidence of postoperative nausea and vomiting. However, ondansetron was significantly more effective in reducing the incidence of vomiting in patients after laparoscopic cholecystectomy than dexamethasone.

## Introduction

Around 30% of individuals undergoing elective procedures under general anesthetic are affected by postoperative nausea and vomiting (PONV). If the patient has risk indicators for PONV, this percentage increases to 70%-80% [1]. The risk factors for early PONV include less than adequate prophylaxis, need for opioid analgesics, longer duration of anesthesia, use of volatile anesthetics, and inadequate analgesia control [2]. The research was carried out to explore how patients perceive postoperative adverse symptoms. The results showed that among 10 symptoms, nausea and vomiting were among the top five least desirable symptoms. Vomiting was considered the most unpleasant symptom, followed by the sensation of gagging on an endotracheal tube [3].

PONV is a heavy economic burden on hospitals, professionals, and patients. Although the cost of antiemetics has gone down, rescue therapy still exhausts funds. The reason for this is likely due to the outcomes of PONV such as increased recovery room stay, length of hospital stay, rehospitalizations, and costs of healthcare staff. For large healthcare centers with a high patient load, this burden is direr as these consequences result in a reduced patient turnover and delays in patient care [4]. Thus, keeping in mind the cost of prophylaxis and patient priority, prophylaxis is routinely recommended and has been shown to increase profits while substantially reducing costs [5].

Severe vomiting postoperatively can cause major complications like dehydration, disturbance in electrolytes, aspiration, incision site disruption, and overall an unpleasant surgical experience [4]. Prophylactic antiemetics before surgery is considered to be the standard protocol to lower the chance of vomiting and nausea after surgery [3]. With a plasma half-life of three hours, dexamethasone is renowned for its efficacy in preventing vomiting and nausea [6]. However, ondansetron, which is a 5-hydroxytryptamine 3 (5-HT3) receptor antagonist has been known to be beneficial for PONV [7]. Ondansetron being a derivative of carbazole is related structurally to serotonin and has 5-HT3 subtype receptor properties that do not alter receptor activity of histamine, dopamine, adrenergic, and cholinergic receptors [8].

Dexamethasone in comparison to ondansetron was shown to be of greater efficacy and more economical in reducing PONV in both a 24- and 3-hour period post-op. The group receiving a 4 mg dose of dexamethasone was also the lowest to require rescue antiemetic therapy in contrast to the 8 mg group and the 4 mg ondansetron group. There was also a decrease in incidence throughout all brackets of time for all groups of patients, demonstrating the positive impact of prophylaxis [9].

It is evident from the literature that prophylactic management of PONV greatly reduces adverse outcomes, lowers the cost burden to healthcare organizations, and significantly increases patient satisfaction [10]. There is no standalone guideline that dictates which drugs are to be used; this decision currently rests upon clinician judgment and patient factors. In light of this, our study aims to quantify the beneficial results of routine antiemetics used within our healthcare system to form informed decisions in patient care. This study may also prompt other such studies within our national healthcare system.

## Materials and methods

A cross-sectional comparative study was undertaken between June 1, 2021, and March 31, 2022, in the Department of Surgery, Civil Hospital, Karachi, Pakistan. All individuals aged between 18 and 70 years who were scheduled for elective laparoscopic cholecystectomy under general anesthesia were considered for inclusion in the study. Pregnant women who had hepatic or renal dysfunction or were taking cortisone or antiemetics before surgery were not allowed to participate.

Each patient participating in this study gave informed consent after the appropriate ethical review committee approved the investigation. Participants were selected according to the aforementioned criteria, then randomly assigned to one of two groups: those who received the drugs during the 30 minutes before anesthesia induction and those who did not.

One dose of ceftriaxone, that is, 1 g was given via intravenous (IV) route while the patients were being given anesthesia. The patients have also been prescribed diclofenac 75 mg via the intramuscular route three times a day on day one of surgery and also when needed. The patients were given the same anesthesia regimen. Propofol was used for induction, isoflurane for maintenance, atracurium for relaxation, and neostigmine for reversal.

The patients were observed for any symptoms such as vomiting, nausea, or the need for any antiemetic medication after the surgery. The evaluation for vomiting and nausea was done in the first 24 hours postintervention. The number of episodes of vomiting and nausea and the length of hospitalization were recorded on the form. Even if vomiting and nausea were present in patients poststudy interventions, dexamethasone 8 mg IV and ondansetron 4 mg IV were given in combination. All study observations were done by trainees to avoid any bias.

Data was examined using the Statistical Package for the Social Sciences (IBM Corp. Released 2019. IBM SPSS Statistics for Windows, Version 26.0, IBM Corp., Armonk, NY, USA). Continuous variables such as age, weight, height, and length of hospital stay were reported as mean and standard deviation. On the other hand, categorical variables such as qualitative data were presented as frequency and percentages. They included patients' gender, sickness, and vomiting. To compare the two groups, the chi-square test was utilized. A *P*-value of 0.05 was regarded as statistically significant.

## Results

During the study period, 259 patients were examined in total - 129 (49.8%) in the dexamethasone group (group A) and 130 (50.2%) in the ondansetron group (group B). The mean age of group A was 42.56 ± 11.9 years, with a mean weight of 61.4 ± 8.5 kg. The mean age of group B was 41.19 ± 10.8 years, with a mean weight of 62.56 ± 6.3 kg. The majority of the patients were females (Table 1). 

**Table 1:** Characteristics of the study participants.

Characteristics	*n* (%)
Age group	
35-45 years	89 (34.4)
45 years and older	170 (65.6)
Gender	
Male	40 (15.4)
Female	219 (84.6)
Drug given	
Dexamethasone	129 (49.8)
Ondansetron	130 (50.2)
Comorbidity	
Diabetes mellitus type 2	106 (40.9)
Hypertension	85 (32.8)

By comparing the effectiveness of each medication in preventing postoperative nausea and vomiting, it was determined that both were equally effective in preventing nausea in the majority of patients (73.85% vs. 65.89%; *P *= 0.162). Ondansetron was significantly more effective than dexamethasone in preventing nausea and vomiting in patients (*P *= 0.004; Table 2).

**Table 2:** Comparison of patient outcomes in ondansetron versus dexamethasone study groups.

Outcome	Ondansetron group (*n *= 130), *n* (%)	Dexamethasone group (*n *= 129), *n* (%)	Total, *n* (%)	*P*-value
Nausea				
Yes	34 (26.15)	44 (34.11)	78 (30.1)	0.162
No	96 (73.85)	85 (65.89)	181 (69.9)	
Vomiting				
Yes	11 (8.46)	27 (20.93)	38 (14.7)	0.004
No	119 (91.54)	102 (79.07)	221 (85.3)	

## Discussion

This study evaluated the impact of dexamethasone and ondansetron on the incidence of postoperative nausea and vomiting in patients who underwent laparoscopic cholecystectomy.

Routine antiemetic prophylaxis minimizes the incidence of surgical nausea and vomiting following discharge. Gupta et al. found that using ondansetron 4 mg IV, ondansetron 8 mg IV, and dexamethasone phosphate 4-10 mg IV reduced the incidence of nausea following discharge. The research indicated that ondansetron medication was more effective than dexamethasone in reducing the likelihood of PONV [11].

The administration of dexamethasone lowers the incidence of postoperative nausea and vomiting following laparoscopic cholecystectomy, as indicated by several earlier investigations [10-12]. Several studies have indicated that there is no significant change in the incidence of PONV when using different combinations of dexamethasone and ondansetron (4 + 4 mg, 4 + 2 mg, 2 + 4 mg, and 2 + 2 mg) in gynecologic laparoscopy (5%, 4%, 9%, and 8%, respectively) [12].

A randomized, placebo-controlled experiment evaluated the effectiveness of dexamethasone, granite trim, and ondansetron in reducing vomiting and nausea in laparoscopic cholecystectomy patients. The incidence of postoperative nausea and vomiting was lowest (25%) with dexamethasone and greatest (75%) with placebo. The incidence of ondansetron was 35% and granisetron was 30%. The variability between ondansetron, granisetron, and dexamethasone was not statistically significant, and PONV was found to be less frequent significantly in groups that were given antiemetics (*P *< 0.05). Similarly, Erhan et al. discussed giving dexamethasone prophylactically greatly reduced PONV in patients who underwent laparoscopic cholecystectomy. In their study, dexamethasone had an equal effect on the patients as did ondansetron and granisetron; however, it was found to be more effective as compared to placebo [13]. 

In their clinical trial, Bano et al. evaluated the effects of mixing ondansetron and dexamethasone with dexamethasone alone in patients undergoing laparoscopic cholecystectomy for PONV. In addition, the combination of dexamethasone and ondansetron was linked with a lower incidence of vomiting and nausea than dexamethasone alone (*P *= 0.035). Nonetheless, the use of rescue antiemetic medications was significantly higher in the dexamethasone groups than in the other groups (*P *= 0.022) [14].

Combining dexamethasone and ondansetron is an effective strategy for lowering the incidence of PONV in patients with laparoscopic cholecystectomy. This combination therapy may provide better control of PONV compared to using either medication alone and may also reduce the need for rescue antiemetic medication [15-17]. Nevertheless, additional research is required to determine these drugs' appropriate dosage and administration schedule. Notably, the usage of any drug should be evaluated by a physician on a case-by-case basis, taking the patient's medical history and any contraindications into account.

Our findings were comparable to those of earlier trials on ondansetron and dexamethasone. Ondansetron is more efficacious than dexamethasone in the preventive treatment of PONV before surgery in laparoscopic cholecystectomy patients. Maitra et al. performed a meta-analysis to compare the efficacy of dexamethasone and ondansetron in reducing the incidence of PONV [18]. Dexamethasone dramatically reduced the incidence in the first four to six hours following surgery, whereas ondansetron had no such effect. Due to heterogeneity, care was advised with interpretation [18]. This contradicts our findings, which suggest that ondansetron was more efficacious. 

While our study has provided valuable insights, it also has limitations that should be considered when interpreting the results. The presence of confounding variables in our study made it challenging to determine the precise impact of the independent variable on the dependent variable. As a result, we suggest that further investigation is necessary to gain a better understanding of the topic.

## Conclusions

The study showed that both dexamethasone and ondansetron were effective in reducing the occurrence of postoperative nausea and vomiting. However, the results also indicated that ondansetron was significantly more effective than dexamethasone in treating these symptoms. It is important for clinicians to weigh the potential benefits and side effects of each medication when making treatment decisions for their patients. The findings of this study can also help inform future research on the effectiveness of different medications for managing postoperative nausea and vomiting.
